# Moderating role of depression in the association between leisure activity and cognitive function among the disabled older people

**DOI:** 10.3389/fpubh.2024.1345699

**Published:** 2024-04-12

**Authors:** Haijun Hao, Miok Kim

**Affiliations:** Department of Social Welfare, Jeonbuk National University, Jeonju, Republic of Korea

**Keywords:** leisure activity, cognitive function, depression, disabled older people, moderation model

## Abstract

**Background:**

This study delves into the complex interaction between leisure activities and cognitive function in older people with disabilities, with a particular emphasis on the moderating influence of depression. Despite the well-documented cognitive benefits of leisure activities among the older people, the intricate relationship between depression and the association between leisure activities and cognitive function in this specific demographic has been rarely reported.

**Methods:**

Drawing on data from the 2017–2018 Chinese Longitudinal Healthy Longevity Survey (CLHLS), this study meticulously examined a cohort of 713 participants aged 65–89 years. We constructed a moderation model to examine the impact of leisure activity on cognitive function in older people with disabilities, with depression levels as a moderating variable.

**Results:**

We found a positive association between engagement in leisure activities and cognitive function, highlighting the potential cognitive advantages associated with leisure engagement among disabled older people. However, the present analysis also reveals a significant moderation effect of depression on this relationship, shedding light on the nuanced nature of this association. Specifically, elevated levels of depression emerge as a critical moderator, attenuating the otherwise favorable impact of leisure activities on cognitive function among older people contending with disabilities.

**Conclusion:**

In conclusion, the findings provide a compelling rationale for tailored interventions that comprehensively target both leisure activity engagement and concurrent depression management, effectively fostering improvements in cognitive function among the cohort of disabled older people.

## 1 Introduction

Cognitive function of older people plays a pivotal role in their overall well-being and quality of life (1, 2). Cognitive decline, a common phenomenon in aging, can significantly impact an individual’s ability to perform daily activities and maintain independence (3–5). This decline is even more pronounced among disabled older people, who may already face additional challenges in their daily lives (6, 7), indicating the necessity of understanding factors that can influence cognitive function in this population.

Leisure activities can be defined as activities people engage in during their free time (8), which have long been recognized as beneficial for cognitive health in older adults (9–11). Engaging in intellectually stimulating and socially engaging leisure activities, such as reading, puzzles, and social interactions, has been associated with a reduced risk of loneliness and an improved cognitive function (12–14). These activities provide opportunities for mental stimulation, maintaining cognitive abilities and promoting brain health. However, the relationship between leisure activities and cognitive function may be effected by other factors such as depression (15, 16). Depression is a prevalent mental health condition among older people, known to have detrimental effects on cognitive function. Meanwhile, depression can impair attention, memory, and executive functions, thereby exacerbating cognitive decline in older adults (17). Moreover, depressed individuals may experience reduced motivation and interest in leisure activities, further compromising their cognitive abilities (16).

Despite the recognized benefits of leisure activities on cognitive function and the negative impact of depression, the interaction between these factors among disabled older people has been rarely examined. Investigating the potential interaction between leisure activities and cognitive function, particularly in the context of depression among this vulnerable population, is of paramount importance. Such understanding can serve as a valuable foundation for designing interventions and strategies aimed at enhancing cognitive health and overall quality of life for disabled older people.

### 1.1 Leisure activity and cognitive function

Engagement in leisure activities has emerged as a salient determinant impacting cognitive function among older adults (18–20). Extensive academic efforts have been devoted to exploring the intricate association between participation in leisure activities and cognitive function. For instance, a systematic review and meta-analysis conducted by Yates et al. (21) examined the impact of leisure activities on cognitive function in older adults, and revealed the association between participating in leisure activities and improvements in various cognitive domains, such as attention, memory, executive function, and processing speed. Moreover, engaging in leisure activities appeared to be related to structural changes in the brain and a deceleration in cognitive decline among older individuals. Further supporting these findings, Chapman et al. (22) conducted a comparative study focusing on physical exercise and cognitive function in older adults, and found that regular physical exercise was linked to better cognitive performance. Additionally, cultural and social leisure activities have been associated with cognitive benefits in older adults. Engagement in activities such as reading, writing, art, learning new skills has shown positive effects on cognitive function (23–26). For instance, in a longitudinal study conducted by Yates et al. (21), it was discovered that consistent engagement in intellectually stimulating leisure activities was linked to the sustained maintenance of cognitive function over the long term in older adults. Additionally, the recent LANCET Dementia Commission has identified social isolation as one of the modifiable risk factors for dementia (27). Social isolation can have a significant impact on the cognitive function of disabled older adults. The influence of biological factors is also very important when studying cognitive impairment, for example, how comorbidities such as vascular and neuroinflammatory conditions can trigger the amyloid cascade that leads to cognitive impairment and dementia (28). Secondly, another important aspect is that all these biological factors contribute to the condition of ‘frailty’ (29). This adds to the overall burden on patients, caregivers and the health system, and patients need more attention from medical and healthcare professionals.

Collectively, previous studies have highlighted the positive relationship between leisure activity and cognitive function in older adults (30–32). Engaging in a variety of leisure activities, including physical exercise, cultural pursuits, and social interactions, appears to promote cognitive health and potentially slow down cognitive decline (23–26). However, previous research has been constrained by two primary limitations: Firstly, there has been a paucity of studies focusing on leisure activity and its impact on cognitive function specifically in disabled older people. Secondly, most studies have solely addressed the influence of leisure activities on cognitive function in older adults, disregarding the potential underlying mechanisms of this relationship. To this end, this study was conducted to examine the effects of leisure activity on cognitive function in disabled older people and explore the potential underlying mechanisms of the impact of leisure activities on cognitive function.

### 1.2 Leisure activity, depression and cognitive function

The association between depression and cognitive function has been extensively investigated, revealing a complex interplay between these two constructs (33–35). Research consistently demonstrates that individuals with depression often exhibit cognitive impairments, including deficits in attention, memory, executive function, and processing speed (36, 37). These cognitive deficits and depression may significantly impact daily functioning and quality of life (38). On the other hand, engaging in leisure activities has been shown to have positive effects on mood and cognitive function in older adults, including those suffering from depression (39). Participation in enjoyable and meaningful leisure activities could serve as a potential protective factor against cognitive decline and may alleviate some depression symptoms (9, 40, 41).

Social Engagement Theory (SET) focuses on the impact of individual participation in social interactions on their cognitive and emotional development. According to SET leisure activities may influence depression and cognitive function in disabled older adults (42). In terms of the relationship between leisure activities, depression, and cognitive function, the potential moderating effect of depression should be clearly acknowledged. Depression can influence engagement in leisure activities, as individuals with depression may experience reduced motivation, interest, and pleasure in participating in activities they once enjoyed. Such decreased engagement in leisure activities may further contribute to cognitive decline or exacerbate existing cognitive impairments in individuals with depression.

In summary, depression is correlated with cognitive impairments, which may affect one’s involvement in leisure activities and subsequently have an impact on cognitive function. While engagement in leisure activities has demonstrated positive effects on cognitive function, depression may potentially moderate this relationship. In this case, further research should be carried out to better understand the complex interrelationships between depression, leisure activities, and cognitive function, ultimately informing interventions and strategies to promote cognitive well-being in individuals with depression.

### 1.3 The current study

Herein, the study was conducted mainly to examine the influence of leisure activity on cognitive function in disabled older people while investigating the potential moderating role of depression in this relationship. The level of engagement in leisure activities was assessed, cognitive function was evaluated using various measures, and the presence and severity of depression symptoms among disabled older people were identified. The contribution of this study lies in exploring the interactive effects between leisure activities and cognitive function in a specific population of older people with disabilities. Previous studies have mainly focused on the impact of leisure activities on cognitive function in older people, with little attention paid to the role of depression and this particular group of older adults with disabilities. This study addresses the following two research questions. (1) Is there a significant association between leisure activity and cognitive function among disabled older people? (2) What is the moderating effect of depression on the relationship between leisure activity and cognitive function among disabled older people? Our hypotheses were (1) that higher levels of engagement in leisure activities among disabled older individuals are positively associated with better cognitive function, and (2) that depression moderates the relationship between leisure activity and cognitive function in disabled older individuals, such that a higher level of depression weakens the association between leisure activity and cognitive function. The innovation of this study lies in its focus on the relationship among leisure activity, cognitive function, and depression in disabled older people. This research is significant for understanding the mental health and cognitive function of disabled older individuals. By investigating the moderating role of depression in the association between leisure activity and cognitive function, we can gain a more comprehensive understanding of the complex relationships among these factors.

## 2 Materials and methods

### 2.1 Data and study population

The data utilized in this study were obtained from the Chinese Longitudinal Healthy Longevity Survey (CLHLS 2017–2018), i.e., a longitudinal survey of the older people population in China, conducted by the Center for Healthy Aging and Development Studies (CHADS) of Peking University, which was initially carried out in 1998. From 1998 to 2018, the CLHLS has conducted eight waves, with a gap of 2–3 years. At the same time, the study population consisted of older people aged 65 years and older, as well as their adult children aged 35–64 years, residing in 23 Chinese provinces (32). The CLHLS is an open, nationally representative cohort study that has included >20,000 individuals from 22 provinces in mainland China, with sampling areas covering >85% of the Chinese population. To ensure representative and reliable sample data, CLHLS utilized a multi-stage disproportionate and targeted random sampling method in its sampling design. Participants or proxy respondents (usually a spouse or close family member) were asked to self-report information on sociodemographic factors, including sex, ethnicity, lifestyle, disease history, etc. Proxy interviews were conducted for respondents who were too impaired to directly participate in study interviews. Very few participants had full proxy interviews in any given wave (<1% of those <90 years of age, ≈1.5% of those 90 to 99 years of age, <5% of those 100–105 years of age). Prior research has ruled out substantial bias due to proxy interviews in the CLHLS (43, 44). Furthermore, additional information regarding the CLHLS survey was available in other studies (45), and its data quality was recognized by scholars both domestically and internationally. In the most recent survey, CLHLS sampled 15,874 older people and collected data on their cognitive function, psychological status, and leisure activities. As the focus of the present study was on the older people population aged between 65–89 years, individuals under the age of 65 and age over 89 years were excluded (46). Moreover, the Activities of Daily Living (ADL) scale was utilized to assess the functional limitations of the older people population, and those with an ADL score of 0 were excluded (47). Consequently, the final sample size of the disabled older people population was 713.

### 2.2 Measurements

#### 2.2.1 Cognitive function

Herein, the Chinese version of the Mini-Mental State Examination (MMSE), a widely used assessment tool for testing cognitive status, was used to assess the global cognitive functioning of older adults with disabilities (48). The MMSE consists of 30 items in six domains including Orientation, Registration, Attention, Language, Memory, and Spatial organization. The total score on the MMSE ranges from 0 to 30, with higher scores indicating better cognitive function. Cognitive impairment was hereby defined as a low MMSE score of 18 or less (49).

#### 2.2.2 Leisure activity

Self-reported information for the leisure activity data was collected through in-home interviews. During the survey, participants were queried about eight different categories of leisure activities, including the frequency of their engagement in tai chi, square dancing, socializing with friends, watching TV or listening to the radio, playing cards or mahjong, reading newspapers or books, raising livestock or pets, gardening, and religious activities. Participants reported how often they engaged in each activity as “almost every day,” “at least once a week,” “at least once a month,” “sometimes,” or “never.” For potential hierarchical analyses, the frequency of the activity was redefined and categorized as “1 = never,” “2 = sometimes,” “3 = at least once a month,” “4 = at least once a week,” and “5 = almost every day,” with values of the 5 options ranging from 1 to 5 successively, and the total score from 0 to 40.

#### 2.2.3 Depression

Depression was evaluated by the Center for Epidemiologic Studies Depression Scale (CES-D-10) in this study. The CES-D-10 includes ten items using a four-point metric, ranging from 0 = “Rarely” to 3 = “Most of the time.” The total scores of CES-D-10 range from 0 to 30 with higher scores suggesting more symptoms of depression. An individual is suggested to have depressive symptoms if he/she scores more than 10 in the CES-D-10. Previous research has used CES-D-10 in Chinese people and reported its good validity and reliability (50).

#### 2.2.4 Covariates

Control variables included the demographic variables age, gender, region, marital status, household income, and perceived social stratification. Perceived social stratification was measured by a single sentence, that is, “How would you rate your economic status compared to other people in your neighborhood?” All answers were assigned a score from 1 to 5, ranging from “very rich” to “very poor” (51–53).

### 2.3 Statistical analysis

**Figure 1:**
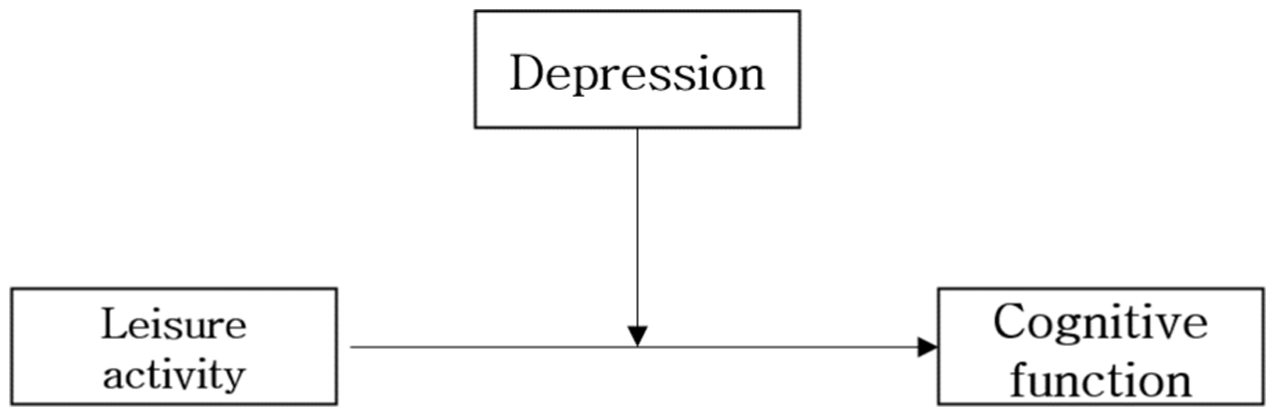
Research model.

The present study was primarily carried out to investigate the effect of leisure activities on cognitive function in disabled older people, and the potential moderating role of depression was also explored. To accomplish this, statistical analysis was conducted using STATA 17 software. Specifically, a three-step analytical approach was employed. Firstly, descriptive analysis was performed to illustrate the general characteristics of the study population, generating a summary of the relevant demographic and clinical variables, such as age, gender, region, and marital status. Secondly, correlation analysis was utilized to examine the relationships between the key study variables, namely leisure activities, depression, and cognitive function, so as to assess the strength and direction of these relationships, and to identify any potential confounding factors that might affect the main study outcomes. Finally, regression analysis was employed to investigate the impact of interaction terms (i.e., moderating effects) on cognitive function, while controlling for potential confounding factors identified during the correlation analysis stage, for which, a series of regression models were constructed to examine the main and interactive effects of leisure activities, depression, and other relevant covariates on cognitive function. Overall, this analytical approach allowed for a thorough examination of the research questions, and provided valuable insights into the role of leisure activities and depression in cognitive functioning among disabled older people. The research model is shown in Figure 1.

## 3 Results

### 3.1 Characteristics of the study population

Table 1 reveals distinct differences between the “65–74” and “75–89” age groups across various aspects. In the realm of health and well-being measures, notable distinctions emerge between the “65–74” and “75–89” age groups. Specifically, the “65–74” age cohort exhibits a higher mean cognitive function score (25.55) compared to the “75–89” age group (22.13), implying superior cognitive abilities within the former group. Additionally, the “65–74” age group presents a greater degree of engagement in leisure activities (mean: 2.15) relative to the “75–89” age group (mean: 1.52). Conversely, in terms of levels of depression, the “65–74” age group reports a slightly higher mean score (7.33) compared to the “75–89” age group (6.62). In summary, the study population is predominantly composed of individuals in the “75–89” age group, accounting for 85.97% of the total, while the “65–74” age group represents 14.03%. While no significant gender differences are observed in perceived social stratification within each age group, the “75–89” age group demonstrates a slightly higher mean score compared to the “65–74” age group. Socio-economically, the “65–74” age group has a higher proportion of rural residents (68%) compared to the “75–89” age group, where there is a higher concentration of urban residents (32%). Additionally, the mean household income is higher in the “75–89” age group, indicating a relatively higher income level in the older age group.

**Table 1:** Characteristics of the study population (*N* = 713).

		65–74		75–89	
		Frequency (*N*)	Percentage	Frequency (*N*)	Percentage
Gender	Male	56	56.00	258	42.09
	Female	44	44.00	355	57.91
Age	Young older people	100 (14.03)			
	Old older people	613 (85.97)			
Region	Rural	68	68.00	386	62.97
	Urban	32	32.00	277	37.03
Marital status	Cohabitation	79	79.00	484	78.96
	Solitary	21	21.00	129	21.04
Household income (Mean/SD) CNY		37082.81 (SD 35798.55)		41347.15 (36462.93)	
Perceived social stratification (Mean/SD)		2.93 (SD 0.77)		3.08 (SD 0.69)	
Cognitive function (Mean/SD)		25.55 (SD 5.46)		22.13 (SD 7.60)	
Leisure activity (Mean/SD)		14.87 (SD 5.77)		12.80 (SD 4.35)	
Depression (Mean/SD)		7.33 (SD 4.96)		6.62 (SD 4.25)	

### 3.2 Characteristics of the main variables

Table 2 provides insights into the distribution and central tendency of the variables measured in the study. Cognitive function scores range from 0 to 30, with an average score of 22.60 and a standard deviation of 7.43. The distribution is negatively skewed and leptokurtic. Leisure activity scores range from 0 to 40, featuring an average value of 13.09 and a standard deviation of 5.80. The distribution is positively skewed and platykurtic. Depression scores range from 0 to 25, with an average score of 6.72 and a standard deviation of 4.36. The distribution is positively skewed and leptokurtic. Overall, the skewness and kurtosis values are within acceptable ranges, indicating normal distributions for data analysis.

**Table 2:** Characteristics of the main variables (*N* = 713).

Variables		Range	Mean	SD	Skewness	Kurtosis
Dependent variable	Cognitive function	0 ~ 30	22.60	7.43	−1.26	4.03
Independent variable	Leisure activity	0 ~ 40	13.09	4.63	1.07	5.80
Moderating variables	Depression	0 ~ 25	6.72	4.36	0.96	4.06

Table 3 presents the associations between cognitive function, engagement in leisure activities, and the onset of depressive symptoms. A cutoff score ≥ 10 was hereby used to identify the respondents exhibiting significant depressive symptoms (54). In terms of leisure activities, compared to their counterparts with depressive symptoms (M = 12.311, SD = 0.392), individuals without depressive symptoms displayed a higher mean score (M = 13.317, SD = 0.192). The corresponding *p*-value of 0.007 demonstrated a statistically significant association between engagement in leisure activities and the onset of depressive symptoms. These findings imply that engagement in leisure activities is linked to the development of depressive symptoms, while there exists no significant association in relation to cognitive function.

**Table 3:** Association between cognitive function, leisure activity and depressive symptom onset.

	No depressive symptoms		Depressive symptoms		*p* value
	Mean	SD	Mean	SD	
Cognitive function	22.739	0.329	22.149	0.498	0.188
Leisure activity	13.317	0.192	12.311	0.392	0.007

### 3.3 Correlation analysis

Herein, a correlation analysis was conducted to examine the relationships among the variables, as shown in Table 4. The results revealed important insights into the associations between cognitive function, leisure activity, depression, and gender. First, in the context of leisure activities, a statistically significant moderate positive correlation coefficient of 0.361 (*p* < 0.001) was observed in relation to cognitive function, suggesting the association between increased engagement in leisure activities and improved cognitive function. The observed correlation coefficient evidences the potential positive impact of leisure activities on cognitive abilities. Next, in terms of investigations into depression, a weak positive correlation coefficient of 0.048 was observed with gender. However, this correlation was not statistically significant (*p* > 0.5), indicating that gender might not exert a substantial influence on depression in this particular study. Overall, these results suggest that other factors may play a more prominent role in determining depression levels, and that gender may not be a significant predictor. In summary, the correlation analysis highlights a significant positive association between cognitive function and leisure activity, supporting the notion that greater participation in leisure activities may contribute to enhanced cognitive abilities. Conversely, the lack of a statistically significant correlation between depression and gender indicates that gender may not necessarily affect the depression levels in this study. These findings offer valuable academic insights into the interrelationships among these variables, emphasizing the potential impact of leisure activity on cognitive function.

**Table 4:** Correlation analysis.

	(1)	(2)	(3)	(4)	(5)	(6)	(7)	(8)	(9)
(1)	1.000								
(2)	−0.138***	1.000							
(3)	−0.242***	0.100***	1.000						
(4)	0.099***	0.006	0.057	1.000					
(5)	−0.014	0.099***	0.018	0.202***	1.000				
(6)	0.101***	−0.019	0.019	0.268***	−0.143***	1.000			
(7)	0.158***	−0.030	0.087**	0.181***	−0.009	0.233***	1.000		
(8)	0.048	0.023	−0.048	−0.017	0.007	−0.026	−0.132***	1.000	
(9)	0.361***	−0.117***	−0.233***	0.172***	−0.035	0.157***	0.133***	0.001	1.000

*p < 0.5; **p < 0.01; ***p < 0.001.

1. Cognitive function, 2. Female (ref. Male), 3. Age, 4. Urban (ref. Rural), 5. Solitary (ref. Cohabitation), 6. Household income, 7. Socio-economic status, 8. Depression, 9. Leisure activity.

### 3.4 The moderating effects of depression

**Figure 2:**
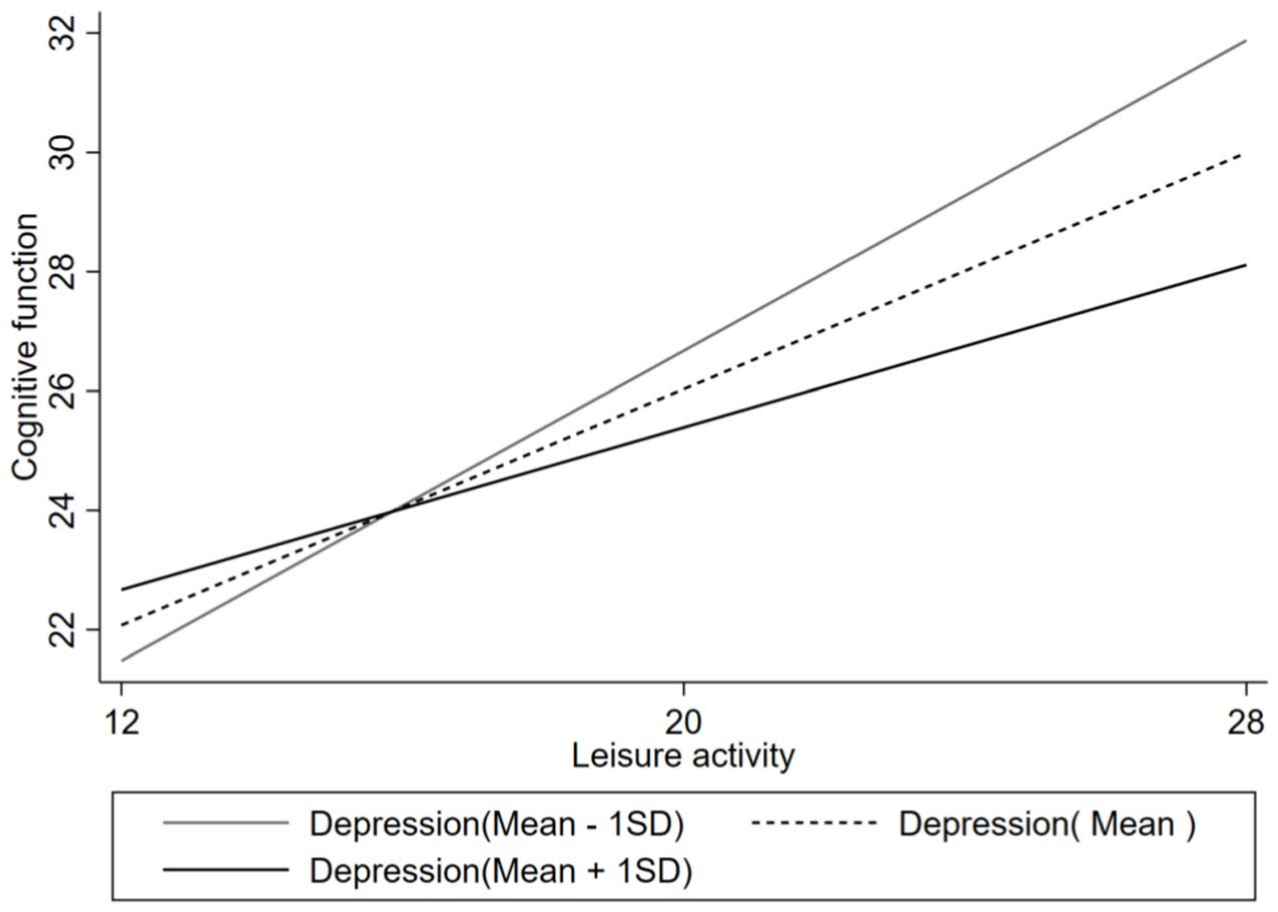
Effects of leisure activity on cognitive function at different depression levels.

Table 5 shows the moderation models, suggesting a moderating role of depression in the relationship between leisure activity and cognitive function. Model 1 controls for age, gender, region, marital status, household income, and perceived social stratification, and the results suggest insignificant effect of depression level on the cognitive function of the impaired older people in this study. With the independent variable leisure activities added, Model 2 explains 19.0% of the dependent variable’s variance (*R*^2^ = 0.190), with an improvement due to the independent variable (Δ*R*^2^ = 0.074), and the overall model is statistically significant (*F* = 20.64, *p* < 0.001). The results indicate that the independent variable of leisure activity has a statistically significant positive effect (*β* = 0.341, *p* < 0.001) on the cognitive function of the impaired older people, suggesting that increased engagement in leisure activities is associated with higher values of the cognitive function. Furthermore, Model 3 includes an interaction term between leisure activity and depression, which exhibits a statistically significant negative effect (*β* = −0.039, *p* < 0.001), suggesting that the relationship between leisure activity and cognitive function is moderated by depression. Specifically, depression moderates the positive relationship between leisure activity and cognitive function among disabled older people (Figure 2).

**Table 5:** The moderating effects of depression.

Variables	Model 1		Model 2		Model 3	
	*B*	*β*	*B*	*β*	*B*	*β*
Female (ref.Male)	−1.676**	−0.112	−1.321*	−0.088	−1.303*	−0.087
Old older people (ref.Young older people)	−0.301***	−0.248	−0.215***	−0.177	−0.217***	−0.179
Urban (ref.Rural)	1.068	0.069	0.453	0.029	0.568	0.037
Solitary (ref.Cohabitation)	0.362	0.020	0.395	0.022	0.523	0.029
Household income	0.194	0.053	0.121	0.033	0.098	0.027
Perceived social stratification	1.679***	0.160	1.277**	0.121	1.189**	0.113
Depression	0.106	0.062	0.114	0.067	0.105	0.061
Leisure activity			0.466***	0.290	0.506***	0.315
Leisure activity × Depression					−0.039***	−0.142
Constant	40.207***		29.172***		29.050***	
*R* ^2^	0.116		0.190		0.209	
Adj. *R*^2^	0.107		0.181		0.199	
Δ*R*^2^	-		0.074		0.018	
*F*	13.17***		20.64***		20.67***	

*p < 0.5; **p < 0.01; ***p < 0.001.

## 4 Discussion

The primary objective of this study was to examine the impact of leisure activity on cognitive function within the disabled older people, with a particular focus on the moderation effect of depression. The results illuminate the intricate interplay among leisure activities, cognitive function, and depression within this vulnerable population.

Consistent with previous research, the present results evidence the positive association between leisure activity and cognitive function in disabled older people (18, 55, 56). Engaging in leisure activities is associated with better cognitive function, suggesting the crucial role of these activities in maintaining cognitive abilities in this population. These findings support the notion that intellectually stimulating, and socially engaging leisure activities can provide cognitive benefits by promoting mental stimulation and brain health. However, this study also reveals the important moderating role of depression in the relationship between leisure activity and cognitive function among disabled older people. Specifically, the beneficial effects of leisure activities on cognitive function are diminished among individuals with higher levels of depression, suggesting the possible role of depression as a barrier that hinders the potential cognitive benefits of leisure activities. The research findings, as described, contribute to the Social Engagement Theory by providing evidence that supports the positive association between leisure activity and cognitive function in disabled older people. The results highlight the importance of engaging in intellectually stimulating and socially engaging leisure activities for maintaining cognitive abilities in this population.

Several explanations can be put forth to understand this moderation effect. First, depression is known to have detrimental effects on cognitive function, including impairments in attention, memory, and executive functions. These cognitive deficits associated with depression may overshadow the positive effects of leisure activities, leading to a reduced impact on cognitive function among depressed individuals (39). Second, depressed individuals may experience reduced motivation and interest in getting involved in leisure activities, thereby terribly limiting their exposure to cognitive stimulation and social interactions, further compromising cognitive function (16). Finally, leisure activities can contribute to the maintenance and improvement of cognitive function in older adults by providing cognitive stimulation, promoting neuroplasticity, and protecting brain health (57). Depression could potentially modulate the influence of leisure activities on cognitive function by affecting brain health.

The present findings have important implications for interventions and strategies aimed at improving cognitive health among disabled older people and indicate the necessity of addressing depression as a potential barrier to the cognitive benefits of leisure activities. Interventions should take into consideration incorporating strategies, such as cognitive-behavioral therapy, integrate mental health and disabled older people care services, or other evidence-based approaches, so as to effectively manage and treat depression. Collaboration between mental health services and older people care providers should be enhanced to ensure a holistic approach in addressing the mental health needs of disabled older people. Coordinated efforts can lead to comprehensive care that considers both cognitive and emotional aspects, thereby improving overall quality of life. By addressing depression, individuals may experience improved cognitive function and better engage in leisure activities, thus creating a positive feedback loop. Additionally, the findings highlight the need for personalized approaches in promoting leisure activities for disabled older people (58). After recognizing the impact of depression, interventions should be tailored to address the unique challenges faced by individuals with high levels of depression. Besides, providing support, encouragement, and creating a supportive social environment may be beneficial in motivating depressed individuals to engage in leisure activities and reap the cognitive benefits (59).

Meanwhile, the present study is also subject to certain limitations. First, the cross-sectional design limits the establishment of causal relationships between leisure activities, depression, and cognitive function. Future longitudinal studies are thus needed to examine the long-term effects and potential bidirectional relationships between these variables. Second, the study relies on self-reported measures, subject to recall bias and social desirability bias. Incorporating objective measures of cognitive function and activity levels should be considered in future research to provide a more comprehensive understanding of the relationship between leisure activities and cognitive function. Lastly, this study is a cross-sectional study, and it is challenging to diagnose cognitive impairment at a specific moment solely based on the 24-point MMSE screening scale. Further analysis is needed. In future research, it is imperative to address the identified limitations by conducting longitudinal studies to explore the long-term effects and bidirectional relationships between leisure activities, depression, and cognitive function. Moreover, incorporating objective measures of cognitive function and activity levels can mitigate recall bias and social desirability bias. Additionally, utilizing more precise diagnostic tools beyond the 24-point MMSE screening scale would enhance the accuracy of diagnosing cognitive impairment at specific moments. By pursuing these avenues, future studies can offer a more comprehensive understanding of the intricate interplay between leisure activities and cognitive function, paving the way for more insightful research in this field.

In conclusion, this study contributes considerably to the growing body of literature on the role of leisure activities in promoting cognitive function among disabled older people. The findings highlight the importance of considering depression as a moderator in this relationship. Addressing depression and designing personalized interventions are crucial steps toward optimizing cognitive health and improving the quality of life for disabled older people. The underlying mechanisms and potential interventions that can effectively promote cognitive function in this population should be continuously explored in future research.

## 5 Conclusion

In conclusion, with a specific focus on the potential moderation effect of depression, the relationship between leisure activity and cognitive function among disabled older people was hereby investigated. The findings provide valuable insights into the complex interplay between leisure activity, cognitive function, and depression in this vulnerable population.

The study results support the notion that leisure activity holds significant potential for promoting cognitive function in disabled older people. Engaging in leisure activities has been shown to have positive effects on cognitive abilities, including memory, attention, and executive function. These activities provide mental stimulation, social interaction, and opportunities for physical exercise, all functioning as important factors contributing to cognitive well-being. Importantly, the study reveals the moderating role of depression in the relationship between leisure activity and cognitive function in disabled older people. The presence of depression symptoms can potentially weaken the beneficial effects of leisure activity on cognitive outcomes, indicating that addressing and managing depression symptoms should be considered an essential component when designing interventions aimed at enhancing cognitive function in this population. Tailored interventions that target both leisure activity engagement and depression management may be necessarily important to optimize cognitive outcomes in disabled older people.

The detailed discussion of the research results can significantly impact future policy-making and practical implementation in several ways. Firstly, the findings emphasize the critical role of addressing depression in disabled older individuals to enhance cognitive function through leisure activities. The proportion of leisure activities in service facilities for disabled older people needs to be increased compared to the current situation. Secondly, the research highlights the need for tailored interventions that consider the moderating effect of depression on the relationship between leisure activity and cognitive function. Policy initiatives can focus on promoting accessible and diverse leisure opportunities that cater to the mental health needs of disabled older people. Service facilities for disabled older people need to pay more attention to investment in mental health programs for older people. Lastly, the study underscores the importance of holistic approaches that address both mental well-being and cognitive health in the care and support services provided to disabled older individuals. By incorporating these research insights into policy development and practical implementation, policymakers can better support the mental and cognitive well-being of disabled older people.

Overall, these findings have important implications for clinical practice and public health interventions. Healthcare professionals and caregivers should recognize the potential benefits of leisure activity in promoting cognitive function among disabled older people. Additionally, depression symptoms should be assessed and addressed as part of comprehensive care plans for this population. Encouraging and facilitating participation in meaningful leisure activities tailored to individual abilities and interests may not only improve cognitive function but also enhance their overall well-being and quality of life.

## Data availability statement

The original contributions presented in the study are included in the article/supplementary materials, further inquiries can be directed to the corresponding author.

## Ethics statement

The studies involving humans were approved by the datasets that support this article are publicly available from the project of the CLHLS. It was approved by research ethics committees of Peking University (IRB00001052-13074). The datasets analyzed during the current study are available online (http://opendata.pku.edu.cn/) from Peking University Open Research Data for researchers who meet the criteria for access to these de-identified data. No experimental interventions were performed. The studies were conducted in accordance with the local legislation and institutional requirements. The participants provided their written informed consent to participate in this study.

## Author contributions

HH: Conceptualization, Writing – original draft, Data curation, Formal analysis, Software, Validation, Writing – review & editing. MK: Funding acquisition, Methodology, Project administration, Supervision, Validation, Writing – review & editing.
